# Partial reprogramming induces a steady decline in epigenetic age before loss of somatic identity

**DOI:** 10.1111/acel.12877

**Published:** 2018-11-18

**Authors:** Nelly Olova, Daniel J. Simpson, Riccardo E. Marioni, Tamir Chandra

**Affiliations:** ^1^ MRC Human Genetics Unit, MRC Institute of Genetics and Molecular Medicine University of Edinburgh Edinburgh UK; ^2^ Centre for Cognitive Ageing and Cognitive Epidemiology, Centre for Genomic and Experimental Medicine Institute of Genetics and Molecular Medicine, University of Edinburgh Edinburgh UK

**Keywords:** aging, aging clock, epigenetic age, iPSC, partial reprogramming, rejuvenation

## Abstract

Induced pluripotent stem cells (IPSCs), with their unlimited regenerative capacity, carry the promise for tissue replacement to counter age‐related decline. However, attempts to realize in vivo iPSC have invariably resulted in the formation of teratomas. Partial reprogramming in prematurely aged mice has shown promising results in alleviating age‐related symptoms without teratoma formation. Does partial reprogramming lead to rejuvenation (i.e., “younger” cells), rather than dedifferentiation, which bears the risk of cancer? Here, we analyse the dynamics of cellular age during human iPSC reprogramming and find that partial reprogramming leads to a reduction in the epigenetic age of cells. We also find that the loss of somatic gene expression and epigenetic age follows different kinetics, suggesting that they can be uncoupled and there could be a safe window where rejuvenation can be achieved with a minimized risk of cancer.

## 1 INTRODUCTION, RESULTS AND DISCUSSION

The human aging process is accompanied by multiple degenerative diseases. Our understanding of such aging related disorders is, nevertheless, fragmented, and the existence and nature of a general underlying cause are still much debated (Faragher, 2015; Gladyshev & Gladyshev, 2016). The generation of induced pluripotent stem cells (iPSCs) allows the reprogramming of somatic cells back to an embryonic stem cell (ESC)‐like state with an unlimited regenerative capacity. This has led to multiple strategies for tissue replacement in degenerative diseases (Takahashi et al., 2007). Clinical application of iPSCs, however, is at its infancy (Singh, Kalsan, Kumar, Saini, & Chandra, 2015; Soria‐Valles et al., 2015; Takahashi & Yamanaka, 2016), and the potency of iPSCs bears risks, not least cancer induction. For example, in vivo experiments with iPSCs have shown that continuous expression of Yamanaka factors (Oct4, Sox2, Klf4 and cMyc, thus OSKM) in adult mice invariably leads to cancer (Abad et al., 2013; Ohnishi et al., 2014).

To avoid this risk, a parallel concept of epigenetic rejuvenation has been proposed: the aging process in cells can be reversed whilst avoiding dedifferentiation (Manukyan & Singh, 2012; Singh & Zacouto, 2010). In other words, an old dysfunctional heart cell could be rejuvenated without the need for it to be passed through an embryonic/iPSC state. The concept of epigenetic rejuvenation requires that rejuvenation and dedifferentiation each follow a distinct pathway. Nevertheless, it is not well understood whether rejuvenation and dedifferentiation are invariably intertwined, or instead whether it is possible to manipulate age without risking dedifferentiation.

The epigenetic rejuvenation potential of partial reprogramming with OSKM factors was previously shown by the forced expression of OSKM+LIN28 in senescent human fibroblasts, which led to recovering the high mobility of histone protein 1β by day 9, a feature characteristic for young fibroblasts (Manukyan & Singh, 2014). Ocampo et al. further demonstrated that partial reprogramming by transient cyclic induction of OSKM ameliorates signs of aging and extends lifespan in progeroid mice, with no resulting teratoma formation (Ocampo et al., 2016). This established partial reprogramming as a promising candidate intervention for age‐related disease. Estimating epigenetic age, which is a promising molecular proxy for biological age (Jylhävä, Pedersen, & Hägg, 2017; Wagner, 2017), was, however, not possible to measure in mice at the time of the Ocampo study. This has left the nature (i.e., dedifferentiation/rejuvenation) of the described cellular changes unexplored:

1. Does the epigenetic remodelling seen truly reflect rejuvenation (i.e., a reduction in cellular/tissue age)? If so, can we observe a decrease in epigenetic age in partially reprogrammed human cells?
2. What is the extent of rejuvenation upon reaching a partially reprogrammed state (e.g., years of epigenetic age decrease)?
3. What are the dynamics of dedifferentiation in early reprogramming?

A major obstacle in understanding the relation between differentiation and aging has been our inability to accurately measure cellular age with a high correlation to the chronological age of the organism. However, over the last five years, a number of age predictors have been developed, the most accurate of which utilize DNA methylation (known as epigenetic clocks) (Hannum et al., 2013; Horvath, 2013; Horvath et al., 2018; Levine et al., 2018; Weidner et al., 2014), with the first Horvath multitissue age predictor being the most widely applicable and used (*r* = 0.96). This “Horvath clock” shows the highest correlation to chronological age, predicting the age (or epigenetic age, eAge) of multiple tissues with a median error of 3.6 years (Horvath, 2013). eAge is distinct from and poorly correlated with other age‐related biomarkers, such as senescence and telomere length, which have been shown to correlate independently with the process of aging (Lowe, Horvath, & Raj, 2016; Marioni et al., 2016). Moreover, an acceleration of epigenetic age as measured by the “Horvath clock” is associated with a higher risk of all‐cause mortality (Christiansen et al., 2016; Marioni et al., 2015; Perna et al., 2016), premature aging syndromes (Down and Werner) (Horvath et al., 2015; Maierhofer et al., 2017), frailty and menopause (Breitling et al., 2016; Levine et al., 2016). All of these studies suggest that eAge may capture a degree of biological aging.

**Figure 1:**
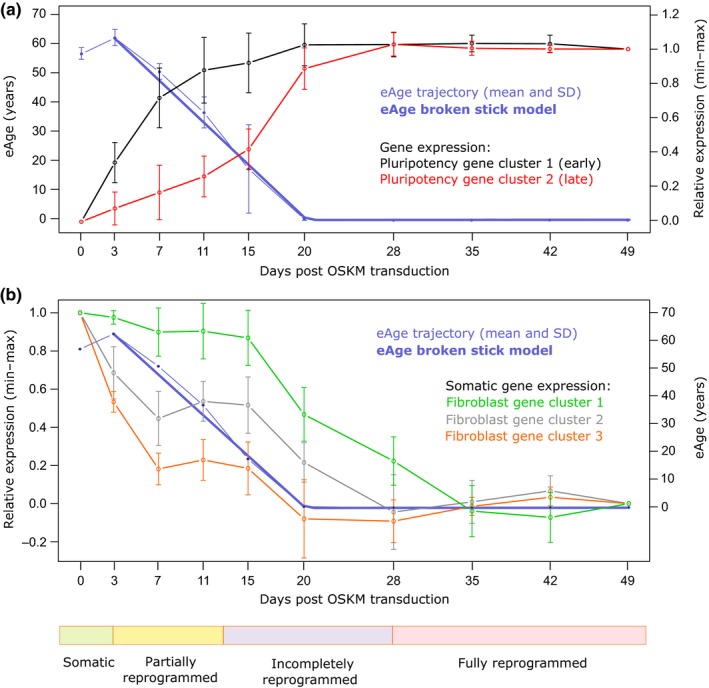
Dynamics of eAge and gene expression in a 49‐day HDF reprogramming time course. (a) Left *Y* axis: eAge trajectory of Horvath's multitissue age predictor calculated from DNA methylation arrays from the following cell populations: day 0 (HDFs), day 3 (OSKM‐expressing EGFP (+) HDFs), day 7, 11, 15, 20 and 28 (human pluripotency marker TRA‐1–60 (+) cells at intermediate stages of reprogramming), and fully reprogrammed iPSCs from days 35, 42 and 49. Data were fit with a broken stick model composed of two linear sections. Error bars represent *SD*. Measured rate (years per day) of eAge decrease [day 3 – day 20] = −3.8, *SE* 0.27, *p* = 3.8 × 10^−7^. Right *Y* axis: Composite gene expression trajectories of key pluripotency markers statistically clustered as per Genolini, Alacoque, and Marianne Sentenac (2015). Microarray expression data were obtained for the same time points and cell subpopulations as for eAge. Relative expression values were log2‐transformed and presented as arbitrary units starting from “0” for “day 0” to “1” for “day 49.” Error bars represent *SD*. (b) Left *Y* axis: Composite gene expression trajectories of key fibroblast markers generated as described for the pluripotency markers in (a). Relative expression values were presented as arbitrary units starting from “1” for “day 0” to “0” for “day 49.” Right *Y* axis: eAge as in (a, left *Y* axis), without *SD*

To understand the dynamics of eAge during reprogramming, we applied Horvath's multitissue age predictor over a previously published reprogramming time course on human dermal fibroblasts (HDFs) (Horvath, 2013; Ohnuki et al., 2014). After OSKM transfection, successfully transformed subpopulations were isolated and analysed at regular time points during 49 days for gene expression and DNA methylation (detailed schematic shown in Supporting Information Figure S1). Epigenetic rejuvenation, that is, decrease in eAge, commenced between days 3 and 7 after OSKM transduction in the partially reprogrammed TRA‐1‐60 (+) cells (characterized in Tanabe, Nakamura, Narita, Takahashi, & Yamanaka, 2013) and continued steadily until day 20, when eAge was stably reset to zero (Figure 1a). A broken stick model (comprising two linear regressions joined at a break point) showed a good fit to the observed data starting from day 3 and measured a steady decrease with 3.8 years per day until day 20 (*SE* 0.27, *p* = 3.8 × 10^−7^) (Figure 1a). The TRA‐1‐60 (+) cell populations at days 7 and 11 have been previously characterized as “partially reprogrammed” for their high expression of pluripotency markers but also high reversion rates towards somatic state (Tanabe et al., 2013). Therefore, the observed eAge decline at days 7 and 11 suggests that partial reprogramming can indeed be considered a rejuvenation mechanism in human cells.

Horvath's multitissue age predictor is the most accurate and widely used for various cell types and tissues (Wagner, 2017). Nevertheless, we calculated eAge from alternative DNA methylation‐based age predictors: four tissue‐specific clocks (Hannum et al., 2013; Horvath et al., 2018; Weidner et al., 2014), one that incorporates clinical measures, called PhenoAge (Levine et al., 2018), and individual CpGs previously correlated with age (Garagnani et al., 2012). All clocks consistently reached the point of reset to their iPSC eAge at day 20, despite the cells not being fully reprogrammed before day 28 (Ohnuki et al., 2014) (Supporting Information Figure S2). Again, eAge showed a steady decline from day 3 to day 20 in the skin and blood and Weidner 99 CpG clocks, PhenoAge declined from day 7 to day 20, whilst the Hannum and Weidner 3 CpG clocks did not produce informative trajectories. Overall, eAge values and “years” of decrease varied between the clocks (actual chronological age of HDF donors is not available for reference) (Supporting Information Figure S2). The highest age associated individual CpG (*ELOVL2*’s cg16867657) showed a similar trajectory to the Horvath eAge decline; however, the remaining CpGs produced inconsistent trajectories (Supporting Information Figure S2). The observed differences are not surprising, given that the alternative clocks were validated for blood (Hannum et al., 2013; Weidner et al., 2014), forensic applications (Horvath et al., 2018), whole organisms (Levine et al., 2018) or various tissues as for the individual CpGs (Garagnani et al., 2012).

In Ocampo et al. partial reprogramming was achieved after just two days of OKSM induction in mice carrying an inducible OSKM transgene (Ocampo et al., 2016). However, such “secondary” systems for direct reprogramming are known to have up to 50‐fold higher efficiency and accelerated kinetics in comparison with virally transduced in vitro systems (Wernig et al., 2008). To facilitate comparison to other systems and associate eAge with intermediate states in the reprogramming trajectory, we compared it to gene expression measured in the same samples. We analysed corresponding microarray expression data for 19 well‐established pluripotency marker genes (Table 1 and Supporting Information Figure S3) as a proxy for reaching a mature pluripotent state (Boyer et al., 2005; Cai et al., 2006; Galan et al., 2013; Ginis et al., 2004; Mallon et al., 2013). We statistically clustered the expression patterns of those genes (Genolini et al. 2015), which resulted in two composite trajectories. These followed previously described expression dynamics of early (cluster 1) and late (cluster 2) activated pluripotency genes (Figure 1a) (Buganim et al., 2012; Chung et al., 2014; Takahashi & Yamanaka, 2016; Tanabe et al., 2013). Pluripotency gene cluster 1 included *NANOG*,* SALL4*,* ZFP42*,* TRA‐1‐60, UTF1, DPPA4* and *LEFTY2*, and their expression increased dramatically within the first 10 days and then established stable pluripotency expression levels by day 20. In contrast, pluripotency gene cluster 2 (containing late expressing genes such as *LIN28*,* ZIC3* and *DNMT3B*) elevated expression more slowly and reached stable pluripotency levels by day 28 (Chung et al., 2014; Tanabe et al., 2013). Interestingly, eAge resets to zero at the same time that the genes in cluster 1 reached their pluripotent state levels, which temporally precedes full pluripotency. This also coincided with a peak in expression of a number of embryonic developmental genes between days 15 and 20, and might suggest that the reset marks a point where the cells reach an embryonic‐like state but are not yet fully pluripotent (Table 1 and Supporting Information Figure S4). In summary, eAge decline is observed well within the first wave of pluripotency gene expression.

**Table 1:** List of pluripotency and fibroblast marker genes used in gene expression clusters

Marker	Gene	Protein name	Accession	Cluster
Pluripotency	*NANOG*	Nanog homeobox	A_23_P204640	1 (early)
Pluripotency	*REX1 (ZFP42)*	Zinc Finger Protein 42	A_23_P395582	1 (early)
Pluripotency	*TRA−1–60/81 (PODXL)*	Podocalyxin	A_23_P215060	1 (early)
Pluripotency	*UTF1*	Undifferentiated embryonic cell transcription factor 1	A_33_P3294217	1 (early)
Pluripotency	*DPPA4*	Developmental pluripotency associated 4	A_23_P380526	1 (early)
Pluripotency	*TDGF1 (CRIPTO)*	Teratocarcinoma‐derived growth factor 1	A_23_P366376	1 (early)
Pluripotency	*SALL4*	Spalt‐like transcription factor 4	A_23_P109072	1 (early)
Pluripotency	*LEFTY1*	Left–right determination factor 1	A_23_P160336	1 (early)
Pluripotency	*LEFTY2*	Left–right determination factor 2	A_23_P137573	1 (early)
Pluripotency	*DNMT3A*	DNA methyl‐transferase 3A	A_23_P154500	1 (early)
Pluripotency	*TFCP2L1*	Transcription factor CP2‐like 1	A_23_P5301	1 (early)
Pluripotency	*TERF1*	Telomeric repeat binding factor (NIMA‐interacting) 1	A_23_P216149	2 (late)
Pluripotency	*DPPA5*	Developmental pluripotency associated 5	A_32_P233950	2 (late)
Pluripotency	*TERT*	Telomerase reverse transcriptase	A_23_P110851	2 (late)
Pluripotency	*ZIC3*	Zic family member 3	A_23_P327910	2 (late)
Pluripotency	*LIN28a*	LIN28 homolog A	A_23_P74895	2 (late)
Pluripotency	*LIN28b*	LIN28 homolog B	A_33_P3220615	2 (late)
Pluripotency	*LECT1*	Leukocyte cell derived chemotaxin 1	A_23_P25587	2 (late)
Pluripotency	*DNMT3B*	DNA methyl‐transferase 3B	A_23_P28953	2 (late)
Fibroblast	*COL3A1*	Pro‐collagen a2(III)	A_24_P935491	1
Fibroblast	*FSP‐1*	Fibroblast surface protein	A_23_P94800	1
Fibroblast	*TGFB3*	Transforming growth factor beta 3	A_23_P88404	1
Fibroblast	*TGFB2*	Transforming growth factor beta 2	A_24_P402438	1
Fibroblast	*COL1A2*	Pro‐collagen a2(I)	A_24_P277934	2
Fibroblast	*ITGA1*	Integrin a1b1 (VLA*‐*1)	A_33_P3353791	2
Fibroblast	*DDR2*	Discoidin‐domain‐receptor*‐*2	A_23_P452	2
Fibroblast	*P4HA3*	Prolyl 4‐hydroxylase	A_24_P290286	2
Fibroblast	*THY1*	Thy*‐*1 cell surface antigen; CD90	A_33_P3280845	2
Fibroblast	*FAP*	Fibroblast activation protein	A_23_P56746	2
Fibroblast	*CD248*	Endosialin, TEM1	A_33_P3337485	2
Fibroblast	*VIM*	Vimentin	A_23_P161190	2
Fibroblast	*COL1A1*	Pro‐collagen a1(I)	A_33_P3304668	3
Fibroblast	*ITGA5*	Integrin a5b1	A_23_P36562	3
Fibroblast	*P4HA1*	Prolyl 4‐hydroxylase	A_33_P3214481	3
Fibroblast	*P4HA2*	Prolyl 4‐hydroxylase	A_33_P3394933	3
Fibroblast	*TGFB1*	Transforming growth factor beta 1	A_24_P79054	3
Fibroblast	*HSP47*	Serpin family H member 1, SERPINH1	A_33_P3269203	–
Fibroblast	*CD34*	Hematopoietic progenitor cell antigen	A_23_P23829	–

Note

Key pluripotent marker genes were selected from Ginis et al. (2004); Cai et al. (2006); Mallon et al. (2013); Galan et al. (2013); Boyer et al. (2005). Fibroblast marker genes were selected from Kalluri and Zeisberg (2006); Zhou et al. (2016); Janmaat et al. (2015); Pilling et al. (2009); Chang et al. (2014); Goodpaster et al. (2008); MacFadyen et al. (2005).

Therapeutic partial reprogramming will depend on rejuvenation with minimal dedifferentiation, which carries the risk of malignancies. We studied the dynamics of fibroblast gene downregulation as a proxy for the loss of somatic cell identity. The individual trajectories of 19 commonly used fibroblast marker genes (Chang, Li, & Guo, 2014; Goodpaster et al., 2008; Janmaat et al., 2015; Kalluri & Zeisberg, 2006; MacFadyen et al., 2005; Pilling, Fan, Huang, Kaul, & Gomer, 2009; Zhou, Yang, Randall Wickett, & Zhang, 2016) (Table 1 and Supporting Information Figure S5) clustered into three composite expression patterns, two of which (clusters 2 and 3) went into an immediate decline after OSKM induction (Figure 1b). However, one fibroblast‐specific cluster (cluster 1) remained stable in its expression for the first 15 days. Interestingly, after day 7, fibroblast‐specific gene expression in clusters 2 and 3 stopped declining and plateaued until day 15, coinciding with a peak in expression of senescence markers between days 11 and 15 (Supporting Information Figure S6). Vimentin (*VIM*), for example, remained at 60% of maximal expression until day 15 of reprogramming, similarly to *FAP*,* CD248* and *COL1A2* in cluster 2 (Supporting Information Figure S5). After day 15, fibroblast gene expression declined rapidly in all three clusters, and only by day 35 had all reached ESC expression levels, marking a complete loss of somatic identity (Figure 1b). Cluster 1, which contains the well‐described indicators of fibroblast identity *FSP1*,* COL3A1* and *TGFB2/3* (Kalluri & Zeisberg, 2006), showed the slowest decline and was also the last to reach ESC expression levels. In summary, we found that a number of fibroblast‐specific genes maintained high expression levels until day 15, by which time a substantial drop in eAge has been observed.

Epigenetic rejuvenation or the reversal of cellular age is a promising concept as it could avoid the oncogenic risks associated with dedifferentiation. Here, we analysed a reprogramming time‐course on HDFs and show that eAge declines in partially reprogrammed cells before their somatic identity is entirely lost.

It is well established that partial reprogramming happens within an early, reversible phase during the iPSC reprogramming time‐course, which involves the stochastic activation of pluripotency genes. It is followed by a more deterministic maturation phase with predictable order of gene expression changes, where cell fate is firmly bound towards pluripotency (Smith, Sindhu, & Meissner, 2016; Takahashi & Yamanaka, 2016). Indeed, it has been shown that mouse fibroblasts fail to become iPSC and revert to their original somatic state if OSKM expression is discontinued during the initial stochastic phase (Brambrink et al., 2008; Stadtfeld, Maherali, Breault, & Hochedlinger, 2008). Previously, Tanabe et al. showed that TRA‐1‐60 (+) cells at reprogramming days 7 and 11 have not yet reached maturation and are partially reprogrammed (Tanabe et al., 2013) but our analysis already shows a decrease in their eAge according to multiple age predictors (Figure 1a and Supporting Information Figure S2). We have also shown that a large proportion of fibroblast marker genes maintain relatively high levels of expression until day 15 (Figure 1b and Supporting Information Figure S5). Nearly, unchanged levels of expression on day 15 were previously also shown for a large proportion of somatic genes (Tanabe et al., 2013). Together with increased senescence gene expression between days 11 and 15 (Supporting Information Figure S6), this likely contributes to the high propensity of partially reprogrammed TRA‐1‐60 (+) cells to revert back to somatic phenotype before day 15 in the time‐course (Tanabe et al., 2013). Interestingly, the stepwise decline of fibroblast gene expression coinciding with a peak in expression of senescence genes seems to delay the loss of somatic identity but not the expression of pluripotency genes. Taken together, the different dynamics between the stepwise fibroblast expression and the linear decline in eAge further indicate that dedifferentiation and epigenetic rejuvenation can be uncoupled.

Our data suggest a window of opportunity within the uncommitted reprogramming phase, where a decline of eAge happens alongside partial maintenance of fibroblast gene expression. A deeper understanding of the kinetics of rejuvenation will be required to master therapeutic partial reprogramming, since any progress of dedifferentiation, even in a small subpopulation, carries the risk of malignancies. Our bulk expression analysis does not allow for a precise definition of the safe rejuvenation boundaries, and further experiments on a single cell level and in in vivo conditions are needed to determine a safe epigenetic rejuvenation window in different reprogramming systems. Upon defining safe boundaries, consideration should also be given to the steep decline of eAge, which resets to zero well ahead of the establishment of a pluripotent state, according to a number of age predictors (Supporting Information Figure S2). Most likely, this marks the point of reaching prenatal or embryonic stage, as suggested by the peak in expression of key developmental genes (Supporting Information Figure S4).

The extent of epigenetic rejuvenation in years (human) or months (mouse), which can be achieved through partial reprogramming, also needs further attention and will most likely differ with the different reprogramming systems. The “Horvath clock” shows up to 10 years of rejuvenation in Ohnuki et al.’s system by day 7 and another 10 + years by day 11. However, the intrinsic median estimation error of 3.6 years in this age predictor, the varying eAge rejuvenation values between the different age predictors and the intrareplicate biological variation seen from the large error bars highlight the need for more experiments and repetitions before this is established with a higher certainty.

Despite the obvious differences in reprogramming kinetics, our results also suggest that the improvements observed by Ocampo et al. in their OSKM‐inducible secondary reprogramming system might be due to epigenetic rejuvenation. It remains to be shown how stable in time the rejuvenated phenotype is in either of the systems. Further analysis is also needed regarding the effect of partial reprogramming on adult stem cells or premalignant cells, which have already shown a higher propensity of transforming to malignancy (Abad et al., 2013; Ohnishi et al., 2014). It is possible that a premalignant phenotype could be attenuated or amplified by partial reprogramming. In summary, our findings reveal exciting possibilities but also open a number of questions and highlight areas that need further attention.

## CONFLICT OF INTEREST

The authors of this paper have no conflict of interests to declare.
