# Supplementary material for: Partial reprogramming induces a steady decline in epigenetic age before loss of somatic identity
Source: Aging Cell. 2018 Nov 18;18(1):e12877. doi: 10.1111/acel.12877 (PMC6351826; doi:10.1111/acel.12877)
Supplement: Supplementary file 1 [file ACEL-18-e12877-s001.pdf]

Figure S1

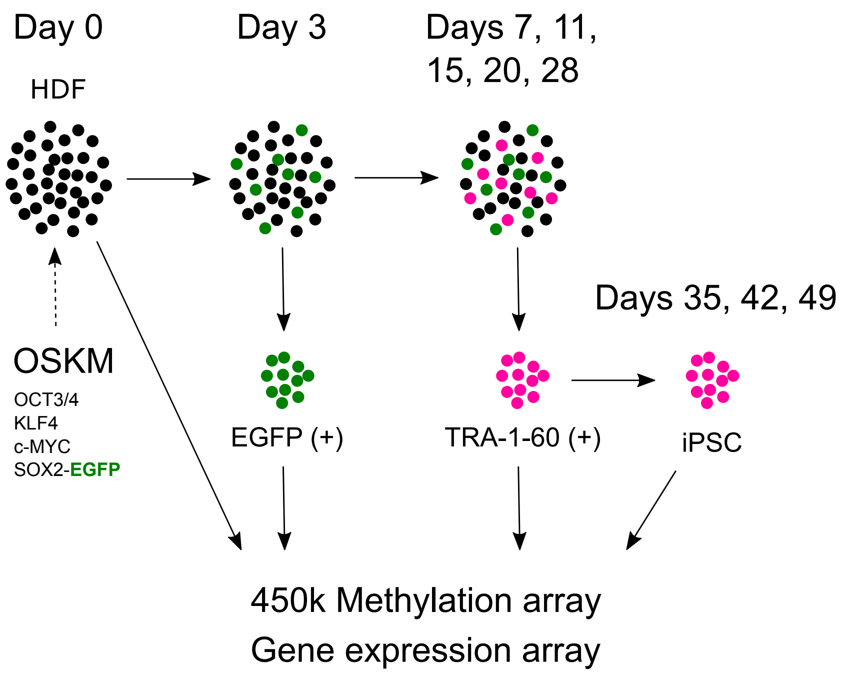

Figure S1. Schematic overview of the experimental setup of Ohnuki et al iPSC time-course and dataset time points. HDF cells were transfected with EGFP-labelled OSKM on day 0 and cultured in virus-containing medium for 24 hours, then replaced by 10%-FBS containing medium for 8 days before replacing with human ESC medium. EGFP (+) cells, representing the population of successfully transfected cells, were sorted by flow cytometry on day 3. Intermediate reprogrammed cells positive for the human pluripotency marker TRA-1-60 were sorted by magnetic activated cell sorting on days 7, 11, 15, 20 and 28 post-transfection. Day 28-sorted TRA-1-60 (+) cells were further expanded and samples collected three more times on each seventh day, i.e. on days 35, 42 and 49. The sorted and collected cells at each time point were subjected to both gene expression and CpG methylation array sequencing. Microarray gene expression was performed for three to four replicates per data point, whilst DNA methylation was performed for two to three replicates per time point. Both 450K DNA methylation array and gene expression microarray datasets were obtained from GSE54848 (Ohnuki et al, 2014).
