# Supplementary material for: Partial reprogramming induces a steady decline in epigenetic age before loss of somatic identity
Source: Aging Cell. 2018 Nov 18;18(1):e12877. doi: 10.1111/acel.12877 (PMC6351826; doi:10.1111/acel.12877)
Supplement: Supplementary file 2 [file ACEL-18-e12877-s002.pdf]

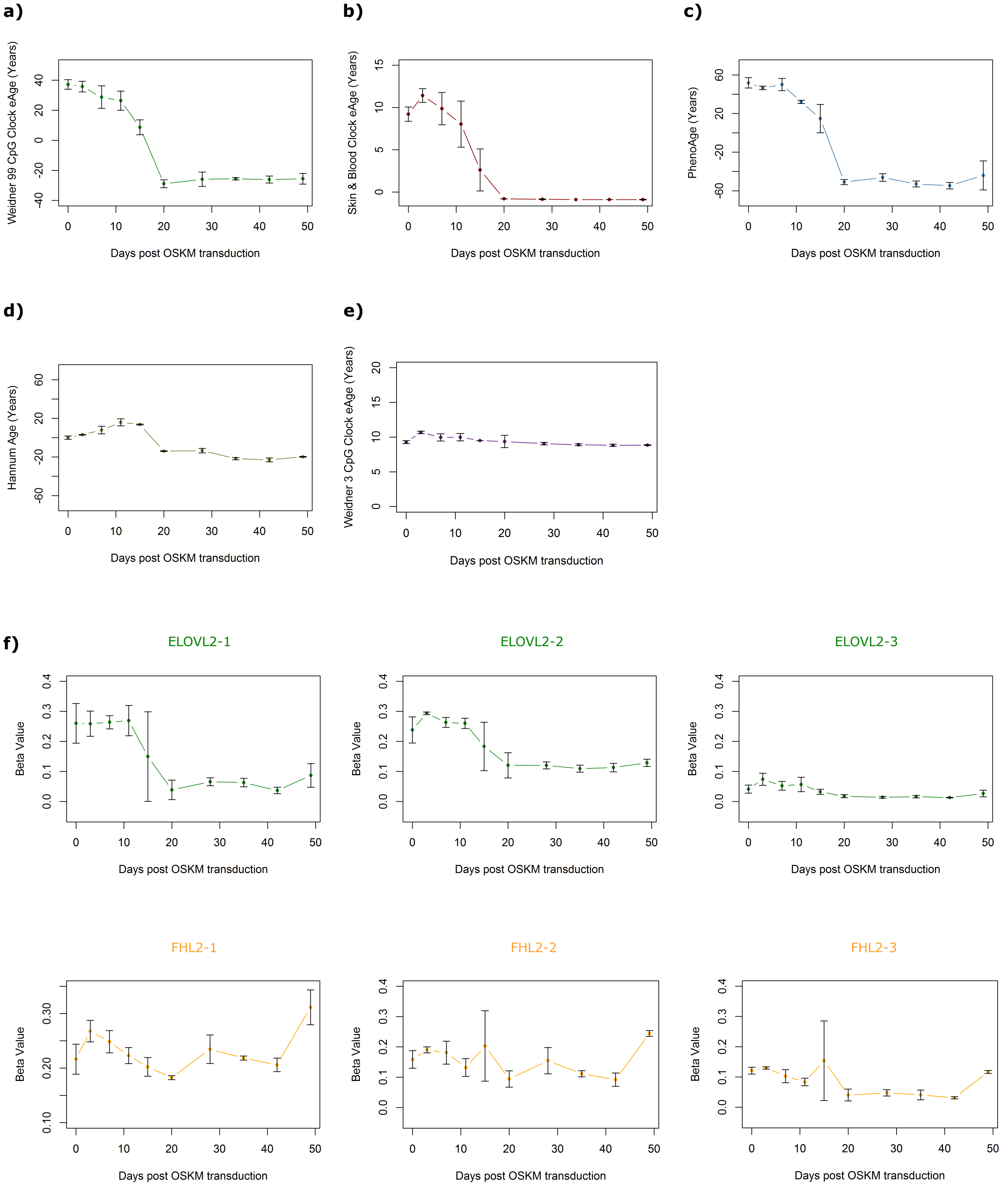

Figure S2. eAge trajectories of different DNA methylation-based epigenetic clocks. (a) Weidner 99 CpG blood-based epigenetic clock (Weidner et al. 2014); **(b)** Skin & blood clock (Horvath et al. 2018); **(c)** PhenoAge (Levine et al. 2018); **(d)** Hannum blood-based epigenetic clock (Hannum et al. 2013); **(e)** Weidner 3 CpG blood-based epigenetic clock (Weidner et al. 2014); **(f)** Individual CpG age predictors found in CpG islands within the *ELOVL2* and *FHL2* genes (Garagnani et al. 2012). Error bars on all plots represent +/- SD from the mean value.
