# Supplementary material for: Partial reprogramming induces a steady decline in epigenetic age before loss of somatic identity
Source: Aging Cell. 2018 Nov 18;18(1):e12877. doi: 10.1111/acel.12877 (PMC6351826; doi:10.1111/acel.12877)
Supplement: Supplementary file 3 [file ACEL-18-e12877-s003.pdf]

a)

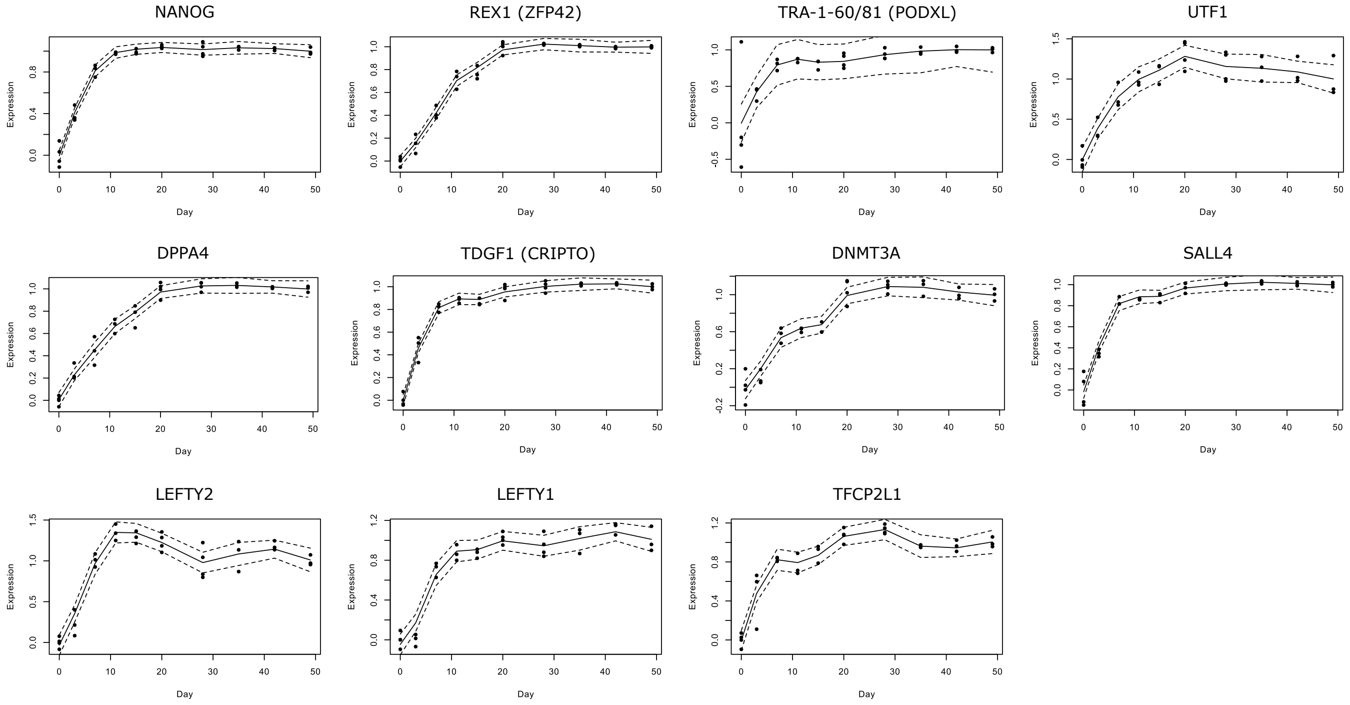

b)

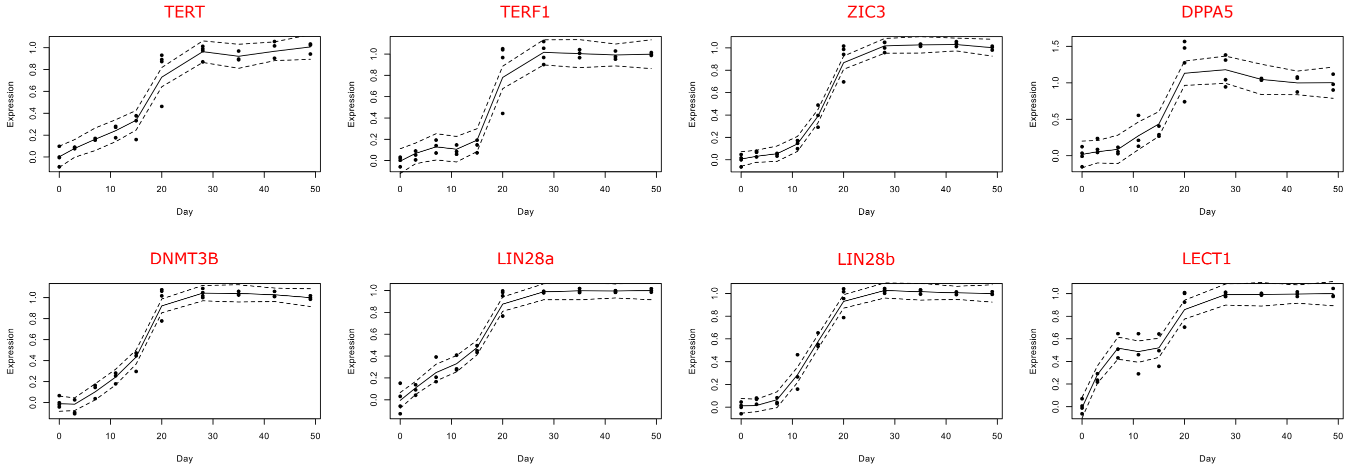

Figure S3. Expression of key pluripotency markers in a 49-day HDF reprogramming time-course. Individual expression dynamics of Cluster 1 genes (early pluripotency markers) in **(a)** and Cluster 2 genes (late expressing pluripotency markers) in **(b)**. Values are LOG2 transformed and normalised between 0 and 1 for 'day 0' and 'day 49', respectively, based on the average values between biological replicates for each time point. Dotted line marks 95% CI. Gene label colours correspond to cluster colours in Fig. 1A.
