# Supplementary material for: Partial reprogramming induces a steady decline in epigenetic age before loss of somatic identity
Source: Aging Cell. 2018 Nov 18;18(1):e12877. doi: 10.1111/acel.12877 (PMC6351826; doi:10.1111/acel.12877)
Supplement: Supplementary file 5 [file ACEL-18-e12877-s005.pdf]

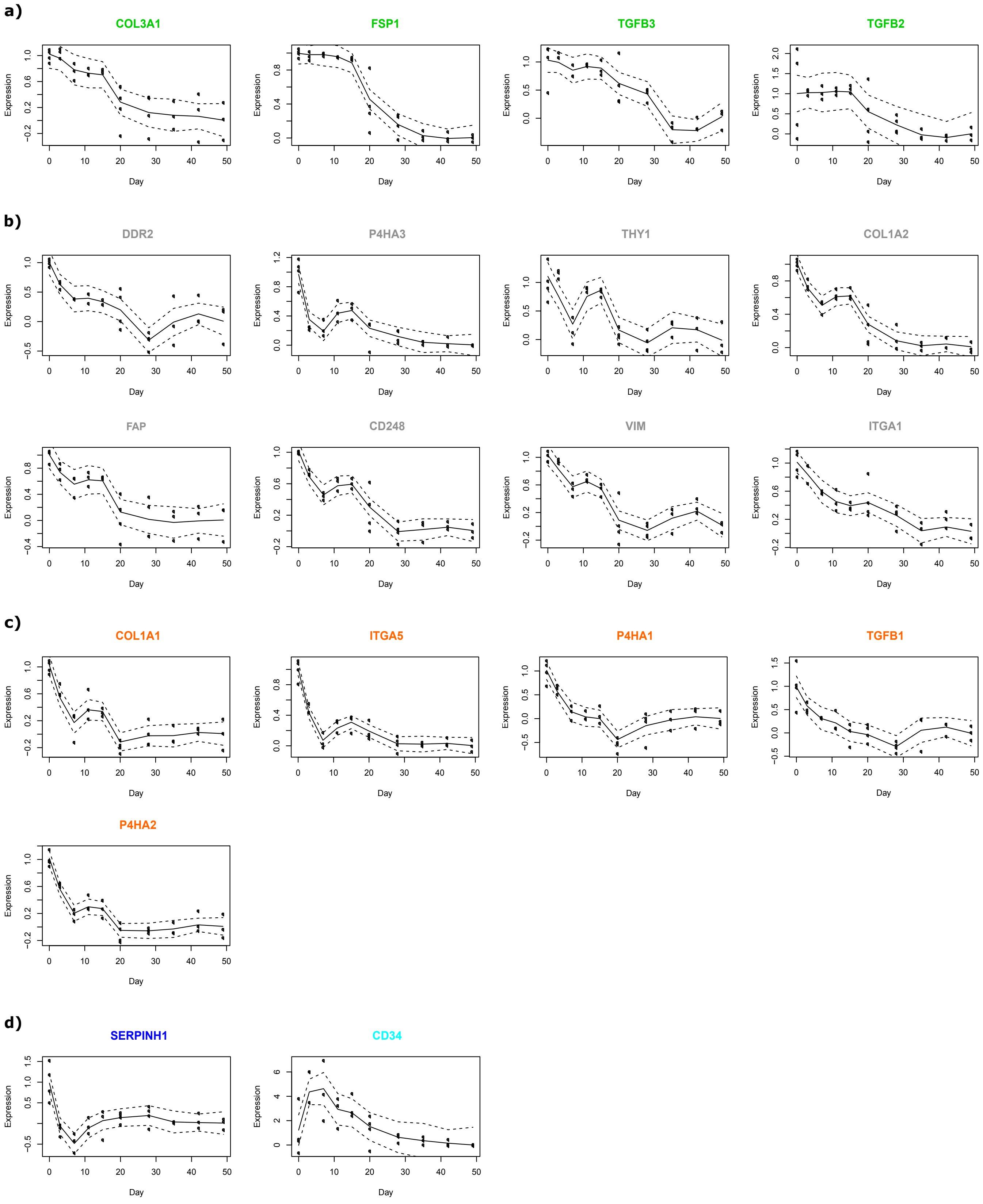

Figure S5. Expression of key fibroblast somatic markers in a 49-day HDF reprogramming time-course. Individual expression dynamics of Cluster 1 fibroblast genes **(a)**, Cluster 2 **(b)** and Cluster 3 genes **(c)**. Values are LOG2 transformed and normalised between 1 and 0 for 'day 0' and 'day 49', respectively, based on the average values between biological replicates for each time point. Dotted line marks 95% CI. Gene label colours correspond to cluster colours in Fig. 1B. SERPINH1 and CD34 expression could not be fit in any of the above clusters and are presented separately **(d)**.
