# Supplementary material for: Partial reprogramming induces a steady decline in epigenetic age before loss of somatic identity
Source: Aging Cell. 2018 Nov 18;18(1):e12877. doi: 10.1111/acel.12877 (PMC6351826; doi:10.1111/acel.12877)
Supplement: Supplementary file 6 [file ACEL-18-e12877-s006.pdf]

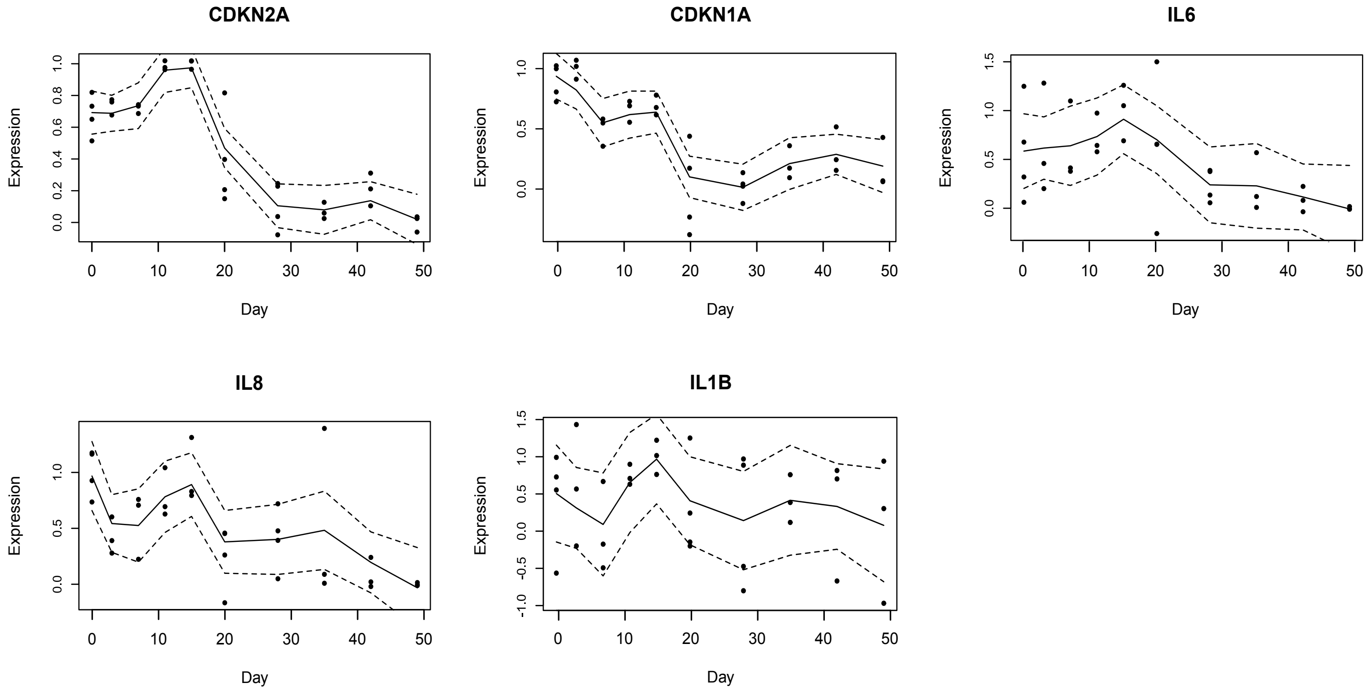

Figure S6. Expression trajectories of key senescence markers in a 49-day HDF reprogramming time-course. Individual expression trajectories of 5 senescence associated genes. Values are LOG2 transformed and normalised between 0 and 1 for the ‘minimum’ and ‘maximum’ value, respectively, based on the average values between biological replicates for each time point. Dotted line marks 95% CI.
