# Supplementary material for: Partial reprogramming induces a steady decline in epigenetic age before loss of somatic identity
Source: Aging Cell. 2018 Nov 18;18(1):e12877. doi: 10.1111/acel.12877 (PMC6351826; doi:10.1111/acel.12877)
Supplement: Supplementary file 7 [file ACEL-18-e12877-s007.docx]

**2** | **SUPPLEMENTAL EXPERIMENTAL**

**PROCEDURES** 8

**2.1** | **Overview of the Ohnuki et al. experimental**

**setup and datasets**

450 K DNA methylation array and gene expression microarray data

of full HDF reprogramming time course were obtained from

GSE54848. A schematic of experimental setup and time points is

provided in Supporting Information Figure S1. Briefly, HDF cells

were transfected with EGFP‐labelled OSKM on day 0 and cultured

in virus‐containing medium for 24 hr, and then replaced by 10%

FBS‐containing medium for 8 days before replacing with human ESC

medium. EGFP (+) cells, representing the population of successfully

transfected cells, which permanently express the OSKM factors,

were sorted by flow cytometry on day 3. Intermediate reprogrammed cells positive for the pluripotency marker TRA-1-60

were sorted by magnetic activated cell sorting on days 7, 11, 15, 20

and 28 posttransfection. Day 28-sorted TRA-1-60 (+) cells were further

expanded and samples collected three more times on each seventh

day, that is, on days 35, 42 and 49. Thus, sorted and collected

cells at each time point were subjected to both gene expression and

DNA methylation array analysis. Microarray gene expression (data

available as LOG2 transformed) was performed for three to four

replicates per data point, whilst DNA methylation data were performed

for two to three replicates per time point.

**2.2** | **Predicting eAge**

The preprocessed 450 K DNA methylation array matrix of average

methylation per CpG site of the full HDF reprogramming time course

was obtained from GSE54848 (downloaded using getGEO function

from GEOquery package) and uploaded to the online DNA methylation

age calculator to assess eAge: https://labs.genetics.ucla.edu/hor

vath/dnamage/(Horvath, 2013). Data processing including Horvath's

normalization was performed according to tutorial guidelines. Missing

CpG values were imputed by Horvath's online DNAm age calculator.

During QC, around 1,600 CpGs were lost; therefore,

methylation data for each time point contained 26,987 CpG sites

out of the suggested 28,587 CpGs, a fact unlikely to have any significant

impact on the normalization or age prediction. PhenoAge, skin

and blood, Hannum, Weidner 99 and 3 CpG age predictors were

applied to average methylation values. Missing CpG values were

imputed as zero before applying these age predictors.

All ages presented in the manuscript are calculated eAges; no

actual ages of HDF donors were available.

**2.3** | **Methylation age trajectories**

For the Horvath multitissue age predictor, a “broken stick” model

with two linear sections was constructed to chart overall change in

DNA methylation age over time between the three HDF cell lines. A

linear mixed model was specified with a random intercept term

for each replicate. A variable break point was set between the minimum

and maximum day, plus and minus a small constant (3 days),

respectively. The predicted values from the regression models were

plotted against the measurement day. For the all other age predictor

plots (Supporting Information Figure S2), mean eAge was calculated

for all samples at each time point (2–3 samples depending on the

time point) and plotted against time during the time course. Standard

deviation for eAge was also calculated and plotted as error bars at

each time point.

**2.4** | **Gene clusters and trajectories**

For each gene in a category (e.g., pluripotent gene list), a loess curve

with a span of 0.5 was fitted with the predicted values extracted at

each time point. The predicted values were then normalized within

each gene to a value of 1 at the first time point and a value of 0 and the last time point (and vice versa for the pluripotent genes).

*K*‐means clustering for longitudinal data was applied to determine

the optimal number of trajectories within each gene category.

All analyses were performed in R, using the kml (Genolini et al.,

2015), lme4 (Bates D, Mächler M, Bolker B, & Walker, 2014) and lmerTest (Kuznetsova, 11

Brockhoff, & Bojesen Christensen, 2016) packages.
